# Reply to Curtis, L. Comment on “Magner et al. Sulforaphane Treatment in Children with Autism: A Prospective Randomized Double-Blind Study. *Nutrients* 2023, *15*, 718”

**DOI:** 10.3390/nu16050674

**Published:** 2024-02-28

**Authors:** Martin Magner, Ivana Švandová, Milan Houška

**Affiliations:** 1 Department of Paediatrics and Inherited Metabolic Disorders, First Faculty of Medicine, Charles University, General University Hospital, 120 00 Prague, Czech Republic; ivasvand@gmail.com; 2 Food Research Institute Prague, 102 00 Prague, Czech Republic; milan.houska@vupp.cz

We thank Dr. Curtis for his interest and feedback [1] on our article “Sulforaphane Treatment in Children with Autism: A Prospective Randomized Double-Blind Study” (2023) [2].

We, in general, agree with Dr. Curtis that the consumption of cruciferous vegetables or phytonutrients like sulforaphane (SFN) may be associated with numerous health benefits and may also potentially interfere with symptoms of patients with neurological conditions like autism. He highlighted that the autistic subject’s consumption of vegetables and fruits is very low. Idiosyncratic food preferences of autistic children are well-characterized by the limited variety of foods and dietary intake, resulting in turn in intestinal microbiome dysregulation, both in terms of quantitative and qualitative abnormalities [3,4,5]. Dr. Curtis’s call for a well-balanced diet rich in vegetables and fruits for ASD children is utterly reasonable, and we fully support it. However, we would be very cautious with the idea of additional significant supplementation with multiple supplements of phytonutrients.

SFN effects and metabolism are heavily studied. A plethora of data has been gained, however, in mouse models or in vitro aerobic or semi-aerobic conditions. Such data are not directly applicable in the context of the effect on the human microbiome. For explain, the effects of sulforaphane have been shown to be altered by the age of mice [6], and cognate thorough studies reflecting the age of ASD patients are missing. Then, less than 3% of bacterial species found within the gut of laboratory mice can be found in humans [7], implicating possible discrepancies with SFN effects in the human gut microbial environment.

Glucosinolate metabolism was shown to exhibit high levels of inter-individual variation for metabolite production in plasma, urine, and stool samples in ASD patients. Personalized differences in microbiome composition are assumed to underly the glucosinolate metabolism variations [8]. Thus, the unified effects of phytonutrient supplementation in the environment of highly variably dysregulated gut microbiome of ASD patients seem to be unlikely. 

Drug-food interactions should also be reflected. Many phytochemicals were identified to interact with drug metabolism and transport. Moreover, they can affect the metabolism of any medication that is a substrate of CYP3A as they alter the gene expression and enzymatic activity of CYP3A. The medication includes anxiolytics, antipsychotics, and/or sedatives [9]. Studies establishing dosage, administration frequency, and other factors ensuring sufficient and clinically significant efficacy with minimal side effects are still enormously scarce.

A balanced diet is, of course, important, and we did the study with the greatest possible desire to help. However, let us be clear—autism is a complex neurodevelopmental disorder, and despite the number of studies with various nutrient supplements, these have come out as mostly ineffective in meta-analyses. It is also hard to find a single molecule that could affect this complex pathophysiology. At the same time, it has a binding effect on families who often cling to insufficiently proven therapies as miraculous, giving them false hope.
